# Net Assimilation Rate Determines the Growth Rates of 14 Species of Subtropical Forest Trees

**DOI:** 10.1371/journal.pone.0150644

**Published:** 2016-03-08

**Authors:** Xuefei Li, Bernhard Schmid, Fei Wang, C. E. Timothy Paine

**Affiliations:** 1 Institute of Evolutionary Biology and Environmental Studies, University of Zurich, Zurich, Switzerland; 2 Department of Physics, Helsinki University, Helsinki, Finland; 3 West China Subalpine Botanical Garden, Institute of Botany, Chinese Academy of Sciences, Dujiangyan, China; 4 Biological and Environmental Sciences, University of Stirling, Stirling, United Kingdom; Chinese Academy of Forestry, CHINA

## Abstract

Growth rates are of fundamental importance for plants, as individual size affects myriad ecological processes. We determined the factors that generate variation in RGR among 14 species of trees and shrubs that are abundant in subtropical Chinese forests. We grew seedlings for two years at four light levels in a shade-house experiment. We monitored the growth of every juvenile plant every two weeks. After one and two years, we destructively harvested individuals and measured their functional traits and gas-exchange rates. After calculating individual biomass trajectories, we estimated relative growth rates using nonlinear growth functions. We decomposed the variance in log(RGR) to evaluate the relationships of RGR with its components: specific leaf area (SLA), net assimilation rate (NAR) and leaf mass ratio (LMR). We found that variation in NAR was the primary determinant of variation in RGR at all light levels, whereas SLA and LMR made smaller contributions. Furthermore, NAR was strongly and positively associated with area-based photosynthetic rate and leaf nitrogen content. Photosynthetic rate and leaf nitrogen concentration can, therefore, be good predictors of growth in woody species.

## Introduction

Growth is of paramount ecological importance for plants, as their survival, reproduction and competitive interactions depend on individual size. Plants vary widely in relative growth rate (RGR) both within and among habitats [1–4], with consequences for community structure and dynamics [5–7]. To determine the mechanistic basis of inter- and intra-specific variation in growth, RGR (in g·g^-1^·day^-1^) can be factored into net assimilation rate (NAR, also called unit leaf rate, g·cm^-2^·day^-1^), specific leaf area (SLA, cm^2^·g^-1^) and leaf mass ratio (g·g^-1^) [8,9] as

RGR=NAR×SLA×LMR (1)

where NAR is the increase in dry biomass per unit leaf area and is a complex physiological variable associated with photosynthetic and respiration rates, SLA is the leaf area per unit leaf mass, a morphological characteristic of plants, and LMR is the ratio of leaf biomass to plant biomass, which reflects a plant’s investment in light capture [9,10].

Previous studies have yielded widely contrasting conclusions regarding the contribution of these factors to RGR. For example, Garnier [11] found that SLA determined RGR, whereas Poorter *et al*. [12] showed NAR to be most important, and Brewster & Barnes [13] found an overwhelming influence of LMR. There are several possible explanations for these differences. First, the relative influence of functional traits on RGR may vary with environmental conditions, especially light availability. If RGR is primarily determined by SLA under low irradiance and by NAR under high irradiance, the compensatory influences of SLA and NAR on RGR between low and high light would limit variation in RGR in heterogeneous light environments [14,15]. Second, SLA, LMR and NAR can all vary over ontogeny [16,17], affecting their contributions to RGR. RGR itself tends to decrease over ontogeny, as ever more biomass is allocated to non-photosynthetic tissue, and respiration costs and self-shading increase [18–20]. Third, studies based on one or few plant species may be unrepresentative of broader patterns because of strong interspecific variation in RGR, NAR, SLA, and LMR [21,22]. Finally, because NAR covaries with absolute growth rate and plant size, size-varying analyses overstate the relationship between NAR and relative growth rate [8]. Size-correction, in which plants are compared at ecologically relevant standardized sizes (or ontogenetic stages), precludes confounding variation in individual size with variation in individual growth rate, yielding a clearer picture of the importance of each component [8,19,23]. Thus, analyses that do not make size-standardized interspecific comparisons on many species suffer from biases that are difficult to quantify [8].

Here, we build upon previous studies by addressing each of these concerns. We assess the physiological basis of variation in growth rates using 14 woody species of subtropical Chinese forests grown for two years over a gradient in light availability. We make comparisons at standardized ages and sizes of planted individuals replicated within each species-by-light combination. We estimate RGR as it varies through time for every individual, which allows us to make size- and age-standardized assessments of the contributions of SLA, NAR and LMR to RGR. Whereas LMR and SLA have easily grasped morphological interpretations, NAR is less concrete. It is what remains of RGR after factoring out the contributions of SLA and LMR. Thus, to better understand the influence of NAR on RGR, we assess the relationships of NAR with in-situ gas-exchange measurements and leaf nitrogen content.

## Materials and Methods

### Study site and species

The study was carried out from August 2007 to July 2009 in an experimental garden in a secondary mixed conifer and broad-leaved subtropical forest near Dujiangyan, southwest China (31°04’ N, 103°43’ E; [24]). No permissions were required to establish the experiment described in this study. The mean annual temperature at Dujiangyan is 15.2°C with an average July temperature of 25°C. The mean annual precipitation of 1341 mm falls mostly between May and October and keeps the annual average relative humidity above 80%. The 14 species used in this study are all woody trees and shrubs common to the forests of the study region. No endangered or protected species were involved in the study. They represent evergreen and deciduous species, and a broad range of shade tolerance and maximal heights (Table 1). Henceforth, they will be referred to by their generic names only.

**Table 1:** Names, characteristics and growth forms of the 14 species examined in this study. Nomenclature follows Flora of China [24].

Species	Leaf habit	Maximum Height (m)	Shade tolerance
*Alangium chinense* (Alangiaceae) (Lour.) Harms	Deciduous	5	Low
*Aralia chinensis* (Araliaceae) L.	Deciduous	7	Intermediate
*Camellia oleifera* (Theaceae) Abel	Evergreen	7	Intermediate
*Castanea henryi* (Fagaceae) (Skan) Rehder & E.H.Wilson.	Deciduous	30	Low
*Choerospondias axillaris* var. pubinervis (Anacardiaceae) (Rehder & E.H. Wilson) B.L. Burtt & A.W. Hill	Deciduous	20	Intermediate
*Diospyros cathayensis* (Ebenaceae) Steward	Evergreen	10	Intermediate
*Diospyros kaki* (Ebenaceae) Thunb.	Deciduous	27	Intermediate
*Lindera communis* (Lauraceae) Hemsl.	Evergreen	5	Low
*Lindera limprichtii* (Lauraceae) H. Winkl.	Evergreen	10	Intermediate
*Phoebe microphylla* (Lauraceae) H.W. Li	Evergreen	10	Intermediate
*Phoebe zhennan* (Lauraceae) S.K. Lee & F.N. Wei	Evergreen	30	High
*Pyracantha fortuneana* (Rosaceae) (Maxim.) H.L. Li	Evergreen	3	Low
*Rhus punjabensis* (Anacardiaceae) J.L. Stewart ex Brandis	Deciduous	10	Low
*Toxicodendron succedaneum* (Anacardiaceae) (L.) Kuntze	Deciduous	10	Intermediate

Seedlings were randomly allocated to one of 15 shade houses, which were, in turn, assigned to one of three light levels. The shade houses had a height of 2.2 m, and an area of 4×5 m. The shade houses were arranged in five spatial blocks, and within each block, light levels were randomly assigned to the three houses. Variation in light availability was created by covering shade houses with layers of neutral shade netting. Instantaneous readings of photosynthetically active radiation (PAR) were made inside and outside each shade house using an SKP 215 PAR Quantum sensor (Skye Instruments Ltd, UK), which indicated that the plants received 43.7±2.1%, 17.1±0.7%, and 2.7±0.1% of full daylight in the high-, medium-, and low-light shade houses, respectively. The 100% light level was achieved by placing potted seedlings in five replicate areas adjacent to the shade houses. Thus, our light-availability gradient spanned the range of light availability in the surrounding forest [25].

### Plant growth

Seeds were collected from beneath parent trees in nearby forests in the spring of 2007 and processed on the day of collection before being germinated under shade cloth in a nursery. In August 2007, seedlings were individually transplanted into plastic pots (30 cm height, 30 cm diameter) filled with farmland topsoil. Two seedlings from each species were placed into each shade house and in each full-light quadrat for a total of 560 plants. Seedlings were separated by at least 0.8 m to avoid shading. Two weeks after transplanting, we replaced seedlings killed by transplantation shock. Plants were watered every 3–4 days or when the soil was dry. Beginning in March 2008, we measured the stem height and basal diameter of each seedling every two weeks for the subsequent two years.

To measure plant functional traits and correlate them with non-destructive measures, 286 and 214 individuals were harvested in August 2008 and July 2009, respectively. After the 2008 harvest, we moved and re-randomized the remaining seedlings among shade houses to reduce the potential for growth responses due to the heterogeneity of light within shade houses. By this time, several *Choerospondias* and *Rhus* individuals had reached a height of 1.5 m in the high light treatment. To avoid nutrient limitation and mutual shading, we transplanted all seedlings from pots into soil.

We used data from the harvests to estimate the aboveground biomass of each individual on the dates that each was measured. The simple linear regression of stem volume against individual biomass was highly predictive (R^2^ = 0.76). As species identity, light treatment and their interaction may influence allometry, species-, light-, and species×light-specific regressions were calculated. Light-specific regressions gave the best estimates (R^2^ = 0.79) and were therefore used to estimate biomass.

The growth of plants can be irregular, owing to the vagaries of environmental conditions and ontogenetic events such as bud break and internode elongation. We used two modeling approaches to obtain accurate estimates of biomass growth rate for each individual. In both, our primary interest lay in obtaining accurate estimates of biomass growth rate for each individual, rather than obtaining the most-parsimonious statistical model. In the mechanistic approach, we predicted daily biomass gain for each plant given its current size, light availability, and the environmental conditions on that day, following the techniques of Turnbull *et al*. [23]. Though this mechanistic modeling approach is conceptually more appealing, as it links growth to time-varying conditions, it did not accurately capture the variation in growth rates among species and light conditions and over time in this case (S1 File). Therefore, we pursued a second, more pragmatic approach. Observation of the growth trajectories indicated that the patterns of growth differed between the two years of the study. Therefore, we split the dataset at November 2008. To evaluate growth rates at the time of the first harvest (August 2008), we used the 14 dates of observation between March and November 2008, whereas growth rates at the second harvest (July 2009) were estimated using the 14 dates of observation between November 2008 and July 2009. We modeled plant biomass as a logistic function of time in the first period because individual growth slowed at the end of the first growing season. We allowed three parameters of the logistic function to vary among species and individuals: size at the beginning of the growing season, the date at which growth was greatest, and the size at the end of the growing season. For harvest two, biomass was modeled as an exponential function of time because we lacked size measurements after the final harvest in July 2009. We allowed two parameters of the exponential function, initial size and relative growth rate, to vary among species and individuals. For both periods, other random-effects structures were tested and they were found to be less appropriate on the basis of Akaike’s information criterion. Biomasses were log-transformed prior to analysis to control heteroscedasticity.

### Trait measurements

Physiological, morphological and biomass-partitioning traits were assessed at each harvest. We used a LI-3100C Area Meter (LI-COR Biosciences, USA) to determine the total fresh leaf area and SLA of each individual. Leaf mass ratio (LMR, %) was calculated as the ratio of leaf biomass to the sum of above- and belowground biomass. Net assimilation rate was calculated by rearranging Eq 1. Nitrogen concentration (N) indicates a plant’s maximum potential photosynthetic rate, given its physiological context [26]. We determined leaf total nitrogen concentration (N_mass_, % dry mass) with a CHN analyzer (Leco CHNS–932, Leco Instruments, USA) in the Institute of Evolutionary Biology and Environmental Studies, University of Zurich, Switzerland. We measured the gas-exchange responses of leaves on a mid-height fully expanded leaf of one plant per species-by-light treatment combination using a Licor 6400 portable photosynthesis system (LI-COR Biosciences, USA). Maximal photosynthetic rate (A_area_, μmol CO_2_·m^-2^·s^-1^) was calculated following photosynthetic rate measurements at 1500, 1000, 750, 500, 300, 150 and 0 μmol m^-2^·s^-1^, holding each light intensity for two minutes. Leaf nitrogen content per area (N_area_, g·m^-2^) was calculated as N_area_ = N_mass_ /SLA. Similarly, A_mass_ (nmol CO_2_·g^-1^·s^-1^) was calculated as A_area_·SLA.

### Dissecting RGR

Testing the correlations between RGR and its components using standard statistical models would be inappropriate owing to a lack of independence (Eq 1; Wright & Westoby 2001). Rather, we decomposed the variance in RGR to calculate the importance of SLA, NAR and LMR [8]. Calculating importance, rather than contribution *sensu* [8] accounts for the covariance among SLA, NAR and LMR, which can mask their contribution to RGR. We discarded observations outside the 95^th^ percentiles of their distributions because estimates of the variance-covariance matrix are sensitive to outliers. As NAR was calculated following Eq 1, a relationship between RGR and NAR could be induced spuriously. Thus, we used half of the individuals sacrificed at the first harvest (n = 128) to predict RGR, and the rest (n = 132) to calculate NAR, SLA and LMR. In this way, RGR and NAR were calculated from independent datasets. Both subdatasets contained all species-by-light combinations and had similar sample sizes in each combination.

We refer to the above analysis as age-standardized, because the growth components were directly calculated from the measurements of mass, and growth rates were predicted for the dates of harvest, when all individuals were the same age (although they varied in biomass). The plant ages in the age-standardized analysis were dictated by the timing of the destructive harvests. To evaluate whether the contributions of growth components depended on plant biomass, we conducted the same analysis at a common plant biomass of 10 g, a biomass attained by all species (although they did so at varying times; S1 Fig). We refer to this as the size-standardized analysis. We estimated leaf mass and leaf area at this reference biomass using regressions between leaf area, leaf mass and total plant mass (R^2^>0.87). Henceforth, we refer to the age-or time-standardized growth rates calculated in these analyses as SGR. All SGRs were calculated as the derivative of the function used to predict growth [19]. Qualitatively similar results were obtained with standardized biomasses of 2 and 20 g [25]; so here we present only the results for 10 g biomass.

Finally, we assessed the physiological bases of variation in growth rates by determining the relationships between SGR and NAR on one hand, and photosynthetic rate and leaf N concentration on the other. Because all of these variables were measured with error, we assessed their relationships using standardized major axis regression. All analyses were performed in the R language and environment version 3.2.2 [27] using the libraries lme4 and smatr [28,29].

## Results

**Fig 1:**
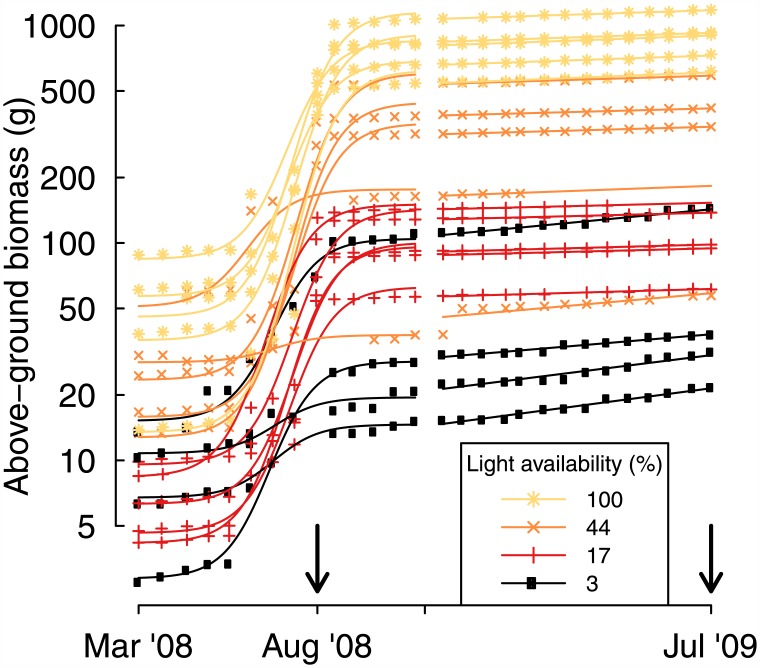
Biomass trajectory for one of 14 studied species, *Choerospondias axillaris* (Anacardiaceae). Until November 2008, growth was modeled as a logistic function of time, whereas afterwards, it was modeled as an exponential function. Vertical arrows indicate dates on which destructive harvests were made and functional traits assessed. Biomass trajectory plots for all species are provided in S1 Fig. Note that the Y-axis is log transformed.

**Fig 2:**
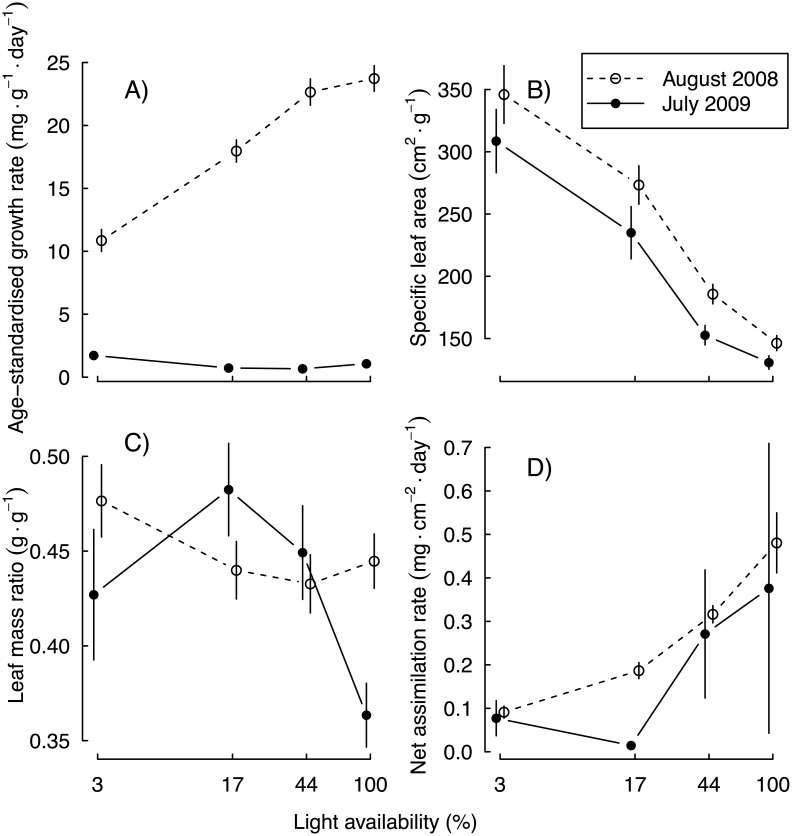
Variation in A) age-standardized relative growth rate (SGR), B) specific leaf area (SLA), C) leaf mass ratio (LMR), and D) net assimilation rate (NAR) over a light-availability gradient. Error bars indicate one standard error of the mean.

The growth models fit the data well, with a root mean square error of prediction of 1.23 g in the first year and 1.06 g in the second, even though individual plant biomass varied over five orders of magnitude during the experiment (Fig 1). Across species and light levels, the mean SGR was 18.8 mg·g^-1^·day^-1^ in year one, but only 1.1 mg·g^-1^·day^-1^ the following year. In year one, SGR increased with increasing light availability, whereas it was insensitive to light the following year (Fig 2A). One species (*Pyracantha fortuneana*), actually lost mass in the lowest light treatment (S1 Fig). SLA decreased slightly from year one to year two, and decreased strongly with increasing light availability (Fig 2B). LMR showed no consistent pattern between years or over the light availability gradient (Fig 2C). NAR, contrastingly, increased with light availability and declined slightly between years (Fig 2D).

### Associations between SGR and its components

**Fig 3:**
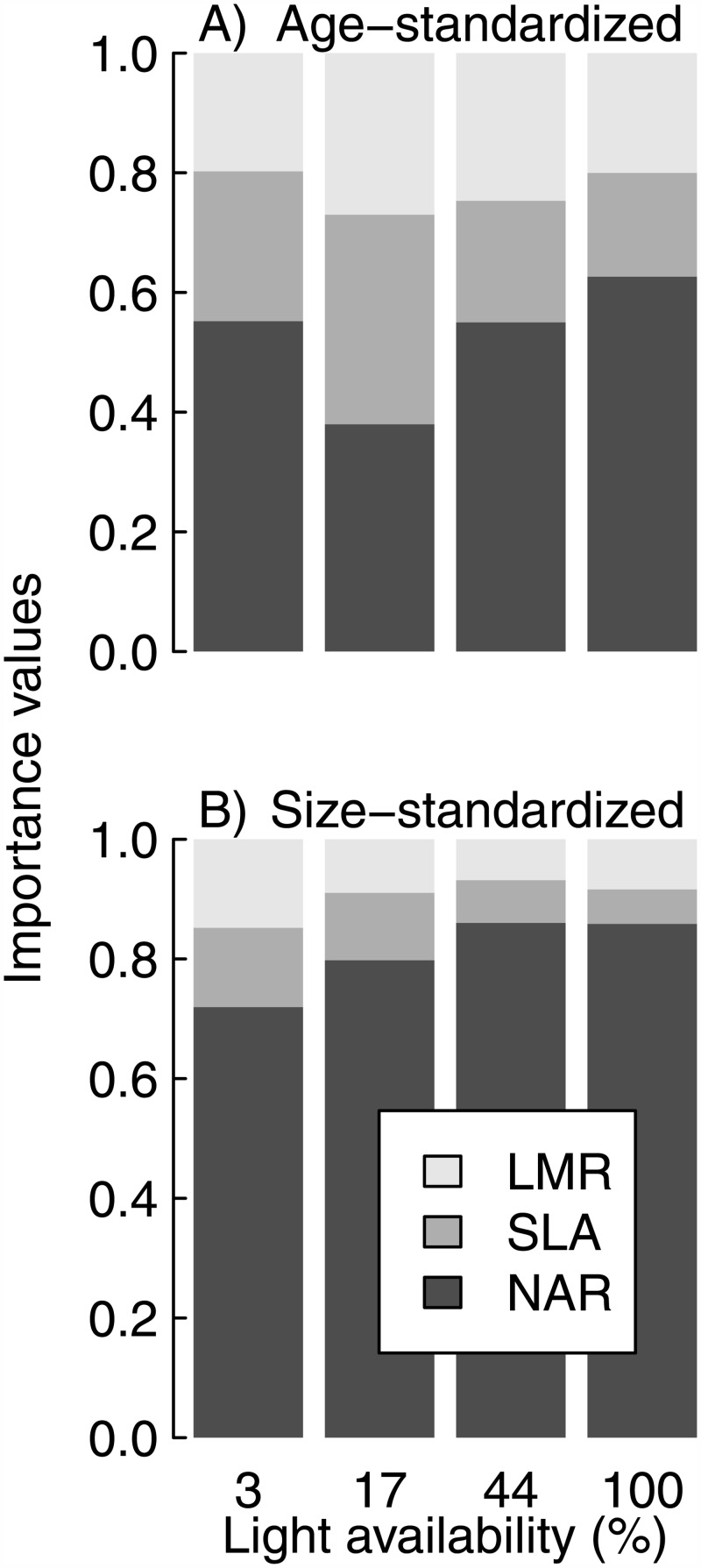
Variation in the importance of each growth component to RGR over a light-availability gradient. A) results from age-standardized analysis, B) results from size-standardized analyses. NAR makes the largest contribution to RGR, regardless of light availability or analysis.

**Fig 4:**
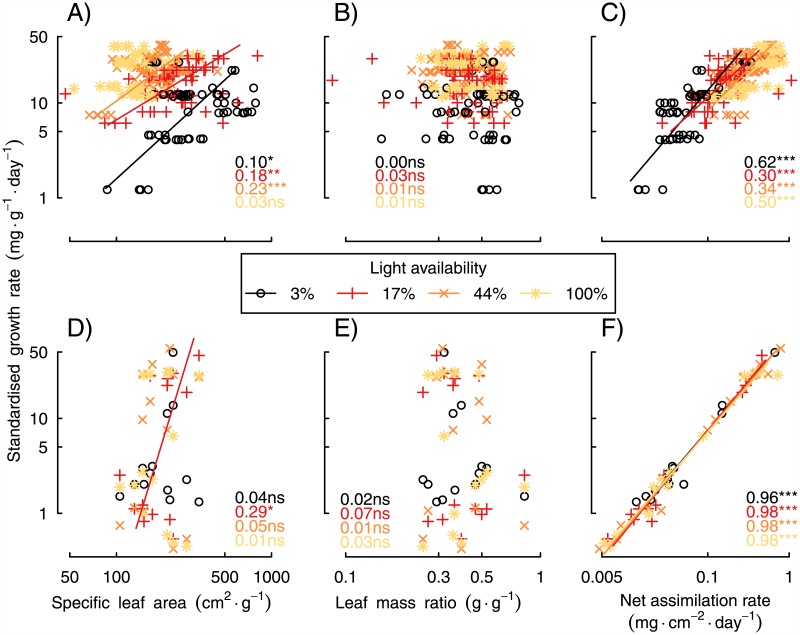
Associations between SGR and its components (SLA, LMR and NAR). A-C) present the age-standardized analysis of data from the first harvest (August 2008), whereas D-F) present the size-standardized analysis, at a standard biomass of 10 g. Statistically significant relationships are shown with solid lines, derived from standardized major axis regression. Numbers show the Pearson correlation coefficient for each light level (*:*P* <0.05, ***: *P* <0.0001, ns: not significant).

Variation in NAR was the most important source of variation in SGR. In both age- and size-standardized analyses, NAR explained at least 38% and 72% of the variation in SGR, respectively (Fig 3). The contributions of SLA and LMR were consistently less than that of NAR, and of similar magnitudes. In the size-standardized analysis, the influence of NAR increased with increasing light availability. SGR was positively correlated with NAR and SLA, whereas it was consistently independent of variation in LMR (Fig 4). In the age-standardized analysis, SGR–NAR relationships were stronger (r ≥ 0.30) than SGR–SLA relationships (r ≤ 0.23), and the latter only arose in the brightest three light treatments. In size-standardized analysis, NAR was even more strongly positively correlated with SGR (r ≥ 0.96), whereas SLA was positively related to SGR only at 17% light availability (r = 0.29; Fig 4D and 4F).

### The physiological correlates of SGR

**Fig 5:**
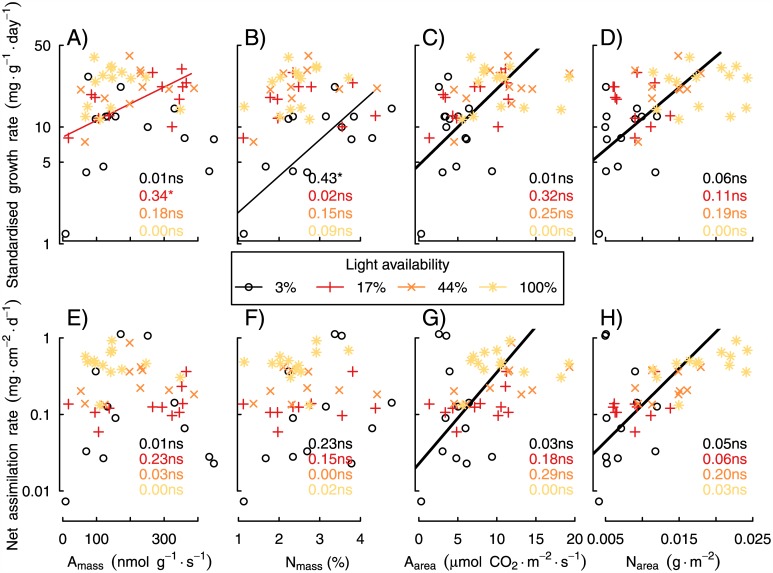
Correlations between SGR and NAR and functional traits, evaluated over a gradient of light availability at the time of the first harvest, when gas-exchange rates were measured. Panels A, B, E and F show functional traits calculated on a biomass basis, whereas C, D, G and H show traits calculated on an area basis. Statistically significant relationships are shown with solid lines, derived from standardized major axis regression. Thick lines indicate relationships over the entire dataset (i.e., disregarding light availability).

To better understand the physiological basis of variation in SGR, we assessed the relationships between SGR and NAR, on one hand, with maximum photosynthetic rate and N concentration, on the other. The associations were much stronger when compared on a leaf-area than on a leaf-mass basis (Fig 5). A_mass_ and N_mass_ were positively associated with SGR, but only at 17% light and 3% light, respectively, whereas they were uncorrelated with NAR. In contrast, A_area_ and N_area_ were strongly positively correlated with SGR and NAR across the entire dataset, i.e., combining the four light treatments. In individual light treatments, however, SGR and NAR were uncorrelated with area-based functional traits, probably because the range of functional traits present in any single light treatment was relatively low.

## Discussion

Net assimilation rate (NAR) was consistently the best predictor of SGR, whereas specific leaf area (SLA) and the allocation of biomass to leaves (LMR) made lesser contributions (Figs 3 and 4). In other words, fast-growing individuals always had high net assimilation rates and individuals with high assimilation rates always grew fast (Fig 4F). Moreover, NAR, and therefore SGR, were strongly positively associated with both maximum photosynthetic rate and leaf N concentration, especially when expressed on the basis of leaf area (Fig 5). Our results are made robust by the many species and wide range of light levels investigated, as well as high frequency of measurements. Furthermore, we used appropriate nonlinear models for growth and made comparisons at standardized ages and sizes [8]. Finally, our results were robust to variation in light availability, plant age and biomass. In the following paragraphs, we discuss their implications for the physiology of plant growth and the potential to predict growth rates.

### Dissecting the components of RGR

Recent meta-analyses showed net assimilation rate (NAR) to be the most important predictor of relative growth rate across a wide range of woody and herbaceous species [30,31]. Meta-analyses cannot be size-standardized. As they rely on summarized published data, they are necessarily “size-varying” analyses. Our age-standardized (and thus, size-varying) analysis confirms their findings (Fig 4C). Recent size-standardized analyses, on the other hand, have come to the contrasting conclusion that variation in specific leaf area (SLA) is the most important contributor to variation in RGR [2,8]. In contrast, the size-standardized analysis of our study showed NAR, rather than SLA, to be the strongest predictor of SGR (Figs 3B and 4D–4F). Our results were obtained with multiple species grown in a single experimental study and thus excluded possible confounding influences that could arise by covariates that vary between or within studies. Such influences are difficult to exclude from meta-analyses. In addition, the duration of our study was substantially greater than that of any similar study, and we examined growth across a wide range in light availabilities, rather than at a single light level. We recommend that more primary data with high temporal resolution from specifically designed experiments be obtained to resolve the contrast between our findings and those of previous studies.

It has been suggested that the relative importance of NAR and SLA on SGR may vary with light availability, with interspecific differences in SLA generating variation in RGR at low irradiance, and interspecific differences in NAR being the main determinant of RGR variation at high irradiance [15]. Shipley’s [30] meta-analysis found support for this hypothesis, though only for herbaceous species, whereas no effect of light on relative importance was observed for woody species. Interestingly, we observed a similar pattern among woody species in our size-standardized analysis (Fig 3B). The change in relative importance along the light availability gradient may be a general phenomenon that simply had not previously been observed in woody species because too few previous studies had been conducted over a wide enough light gradient [30], whereas light availability in the current study ranged from 3–100%.

### Physiological correlates of NAR

By combining detailed physiological measurements with size-standardized analyses, we were able to further dissect the components of NAR, the primary source of variation in RGR. Size-standardization allowed us to interpret NAR as a primarily physiological parameter. As explained above, doing so is problematic in size-varying analyses [8]. We found NAR to be positively correlated with leaf area-based maximum photosynthetic rate and nitrogen concentration, as in some previous studies [32]. Hérault *et al*. [33], however, found no association between leaf mass-based nitrogen concentration and growth rate in Neotropical trees, and Quero *et al*. [34] found RGR to be independent of mass- and area-based photosynthetic rates, though strongly associated with daily carbon gain, among four *Quercus* species. Drawing effective comparisons with previous studies is complicated by the fact that no previous studies, to our knowledge, have simultaneously included size-standardized calculations of growth rate, estimated growth rate and its components using independent sets of individuals, correlated components with physiological rates, and used multiple species in a single experiment with a wide range of manipulated light availabilities. Studies that have assessed growth using nonlinear functions did not measure physiological variables, and those that did assess physiology performed size-varying analyses of growth, exaggerating the importance of NAR. Determining the generality of our finding that NAR is strongly correlated with area-based photosynthetic rate and nitrogen concentration will depend on carefully designed studies that include both aspects.

Consistent with ecophysiological theory, our study shows that area-based traits are better predictors of SGR than are mass-based traits (Fig 5). Regardless of leaf thickness, only a few cell layers are responsible for the bulk of light capture and photosynthesis [35]. Thus, evolution should favor traits associated with photosynthetic function primarily proportional to area [22]. Measuring respiration rates, which was beyond the scope of this study, would allow net carbon gain to be estimated, and may yield further insight into the physiological underpinnings of NAR, and thus RGR [34].

## Conclusions

What generates the large variation in growth rates among co-occurring species and individuals of woody plants? With detailed data on growth dynamics and physiological performance, as well as an appropriate experimental design and analytical technique, we found strong evidence that it is variation in NAR, at least for a set of common woody species from subtropical Chinese forests. NAR, however, is a slippery concept, as it is what remains of RGR once SLA and LMR have been factored out (Eq 1). We therefore also assessed its physiological correlates. NAR, and therefore RGR, were strongly positively associated with leaf area-based photosynthetic rate and N concentration. Our study thus provides a response to Shipley’s [30] exhortation that “to find easily measured attributes that correlate well with NAR.” We show that A_area_ and N_area_ are suitable proxies for NAR, and thus simple, powerful predictors of growth rates for juvenile woody plants.

## Supporting Information

S1 Fig Biomass trajectory for all 14 studied species. Until November 2008, growth was modeled as a logistic function of time, whereas afterwards, it was modeled as an exponential function. Vertical dotted lines indicate dates on which destructive harvests were made and functional traits assessed. Note that the Y-axes are log transformed.
(PDF)

S1 File Details of the mechanistic model of growth. (DOCX)
