# Supplementary figures and images for: Net Assimilation Rate Determines the Growth Rates of 14 Species of Subtropical Forest Trees
Source: PLoS One. 2016 Mar 8;11(3):e0150644. doi: 10.1371/journal.pone.0150644 (PMC4783115; doi:10.1371/journal.pone.0150644)

Supplemental Figure 1

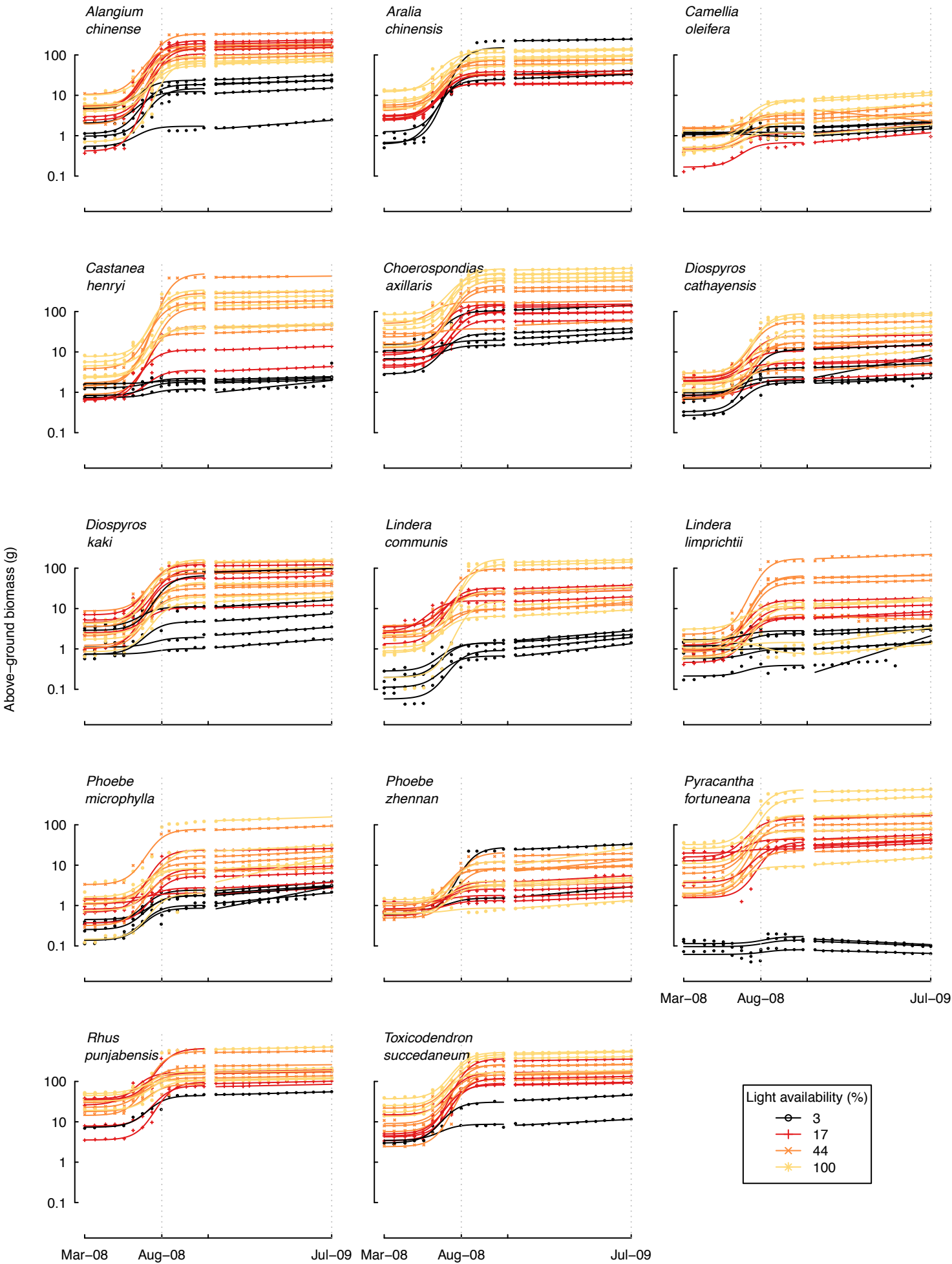

Supplement: S1 Fig — Until November 2008, growth was modeled as a logistic function of time, whereas afterwards, it was modeled as an exponential function. Vertical dotted lines indicate dates on which destructive harvests were made and functional traits assessed. Note that the Y-axes are log transformed. (PDF) [file pone.0150644.s001.pdf]
