# Supplementary material for: Net Assimilation Rate Determines the Growth Rates of 14 Species of Subtropical Forest Trees
Source: PLoS One. 2016 Mar 8;11(3):e0150644. doi: 10.1371/journal.pone.0150644 (PMC4783115; doi:10.1371/journal.pone.0150644)
Supplement: S1 File — (DOCX) [file pone.0150644.s002.docx]

**Appendix 1: Details of mechanistic model of growth**

Plants often have irregular growth rates, reflecting the vagaries of environmental conditions such as temperature and light. Such temporally-varying predictors are not easily incorporated into classical analyses of plant growth, rooted as they are in the statistics of ordinary least squares. To allow for such variations, we used a mechanistic growth model that predicted the daily growth of each plant given its current size, light availability, and the environmental conditions on that day (Turnbull *et al.*, 2008). Since plants become increasingly inefficient in terms of biomass growth when they grow larger, we developed our growth model based on a power-law function (Paine *et al.*, 2011). The daily environmental conditions temperature and light were incorporated in the following way. For a given plant *i* belonging to species *s* on day *d* after the start of the experiment, we calculated daily biomass gain as:

$C_{isd}=T_{isd}\times L_{is}\times G0_{s}\times M_{isd}^{\beta_{s}}$ (Equation 1)

Here *G0_s_* is a growth constant, *M_isd_* is the whole-plant biomass and *β*_s_ governs the rate of slowing in biomass accumulation as biomass increases. *T_isd_* adjusts growth according to daily temperature and *L_is_* adjusts growth according to light availability. Daily temperature affects biomass gain via a three-parameter logistic function:

$T_{isd}=1+ \frac{Tcp_{s}-1}{1+e^{Tobs_{d}-Tmid_{s}}/Tsc_{s}}$ (Equation 2)

As the observed temperature *Tobs* increases, *Tisd* approaches 1, thus temperature does not reduce biomass gain. Contrastingly, as *Tobs* decreases, *Tisd* approaches the lower horizontal asymptote (*Tcp)*, incorporating winter-time of reduction in growth. *Tmid* is the inflection point, indicating the temperature at which *Tisd* is halfway between the asymptotes. *Tsc* indicates the temperature at which *Td* is roughly three-quarters of the distance between the asymptotes. Daily temperatures were obtained from a thermometer buried in the soil of one shadehouse. Because there were no measurements available before day 58, we assigned to these days a temperature of 22.46°C, the mean soil temperature during the same period of time in the second year.

Daily biomass gain is taken to be an asymptotic function of light availability. As light availability approaches 100% (full daylight), *Ld* approaches 1:

$L_{is}=1-e^{(L\min_{s}-Lobs_{i})/L0.5_{s}}$ (Equation 3)

where *Lmin* indicates the light compensation point (the minimum amount of light needed to maintain carbon balance). *L0.5* represents the light level at which *Lid* = 0.5, and thus indicates low-light growth efficiency.

We calculate RGR by combining equations 1 and 3:

$$RGR_{isd}=C_{isd}/M_{isd}=T_{isd}\times L_{is}\times G0_{s}\times M_{isd}^{\beta_{s}-1}$$

As RGR calculated in this way is corrected for plant biomass, we referred to it size-standardized RGR (SGR).

**Parameter estimation**

The model required the estimation of seven parameters: *G0, β, Tcp, Tmid, Tsc, Lmin* and *L0.5*. To define the model and estimate these parameters, we used “Filzbach”, a packaged C library (Turnbull et al. 2008), which applies Markov Chain Monte Carlo (MCMC) methods to generate a posterior distribution for each parameter given model and the data. Bayesian posterior estimates numerically resemble much the estimates from maximum or restricted maximum likelihood methods (Kéry, 2010, Link & Barker, 2010), but they are exact rather than approximate, because they account for the full uncertainty in the modeled system (Gelman & Hill 2007). We ran the model for sufficient iterations (up to 200,000) that the likelihood of the parameters, given the model and data, were constant. The uncertainty in the parameter estimate was evaluated using the posterior distribution of this parameter. We report here the mean and 95% Bayesian credible interval for each parameter (analogous to a 95% confidence interval in classical statistics) in Table 2.

To determine the degree to which each parameter varied among species, we compared the Bayesian information criterion (BIC) (Schwarz, 1978) from model fits where the parameters were made species-specific or global. We chose BIC as the criterion for model selection as BIC yields more parsimonious models for large datasets than does AIC (Burnham & Anderson, 2002). We began with a model in which all seven parameters were species-specific, and then fitted models with each parameter in turn made global. All of these models had lower BICs compared with the fully species-specific model. From the set of the seven reduced models we selected the one with the lowest BIC and set the relevant parameter to be permanently global. We then fitted the six possible models with a second global parameter. From these six models we again chose the one with the lowest BIC, fixing the corresponding global parameter, and so on until the BIC of the selected model no longer decreased. The resulting model was then used as the most parsimonious fit. Despite the simplifications, the resultant mechanistic models predicted biomass poorly (compare Appendix Figure 1, below, with Figure 1 and Supplemental Figure 1). Therefore, we used nonlinear mixed effect models to predict biomass and growth rates, as described in the main text.

**Literature Cited**

Burnham, K.P. & Anderson, D.R. (2002) *Model Selection and Multimodel Inference: A Practical Information-Theoretic Approach.* Springer-Verlag.

Gelman, A. & Hill, J. (2007) *Data Analysis Using Regression and Multilevel/Hierarchical Models.* Cambridge University Press, Cambridge, UK.

Kéry, M. (2010) *Introduction to WinBUGS for Ecologists– A Bayesian approach to regression, ANOVA, mixed models and related analyses.* Academic Press, Burlington, MA.

Paine, C. E. T., T. R. Marthews, D. R. Vogt, D. Purves, M. Rees, A. Hector, and L. A. a. Turnbull. 2012. How to fit nonlinear plant growth models and calculate growth rates: an update for ecologists. Methods in Ecology and Evolution 3:245–256

Turnbull, L. A., C. Paul-Victor, B. Schmid, and D. W. Purves. 2008. Growth rates, seed size, and physiology: do small-seeded species really grow faster? Ecology 89:1352–63.

**Appendix Figure 1**

Biomass predictions for each species under each light treatment (3%, 17%, 44% and 100%) from mechanistic growth model (solid line) with 95% confidence interval (grey shading). Dots indicate observed biomasses.
